# Surface contact and design of fibrillar ‘friction pads’ in stick insects (*Carausius morosus*): mechanisms for large friction coefficients and negligible adhesion

**DOI:** 10.1098/rsif.2014.0034

**Published:** 2014-05-06

**Authors:** David Labonte, John A. Williams, Walter Federle

**Affiliations:** 1 Department of Zoology, University of Cambridge, Cambridge CB2 1TN, UK; 2 Department of Engineering, University of Cambridge, Cambridge CB2 1TN, UK

**Keywords:** tribology, fibrillar adhesives, controllable attachment

## Abstract

Many stick insects and mantophasmids possess tarsal ‘heel pads’ (euplantulae) covered by arrays of conical, micrometre-sized hairs (acanthae). These pads are used mainly under compression; they respond to load with increasing shear resistance, and show negligible adhesion. Reflected-light microscopy in stick insects (*Carausius morosus*) revealed that the contact area of ‘heel pads’ changes with normal load on three hierarchical levels. First, loading brought larger areas of the convex pads into contact. Second, loading increased the density of acanthae in contact. Third, higher loads changed the shape of individual hair contacts gradually from circular (tip contact) to elongated (side contact). The resulting increase in real contact area can explain the load dependence of friction, indicating a constant shear stress between acanthae and substrate. As the euplantula contact area is negligible for small loads (similar to hard materials), but increases sharply with load (resembling soft materials), these pads show high friction coefficients despite little adhesion. This property appears essential for the pads’ use in locomotion. Several morphological characteristics of hairy friction pads are in apparent contrast to hairy pads used for adhesion, highlighting key adaptations for both pad types. Our results are relevant for the design of fibrillar structures with high friction coefficients but small adhesion.

## 1. Introduction

Attachment devices for climbing have evolved in many insect orders [1]. When used during locomotion, these structures have to meet challenging demands: attachment forces must be strong enough, but detachment has to be fast and energy efficient [2]. In addition, plant surfaces can vary strongly in their surface topography and chemistry [3]. Thus, insects have to be able to attach to substrates with diverse properties, ranging from smooth to rough and from hydrophilic to hydrophobic. In order to cope with these challenges, insects make use of a ‘division of labour’ between different types of tarsal attachment structures [4–8].

For example, insects use claws on sufficiently rough surfaces, and use specialized soft attachment pads when the claws cannot grip [5]. These pads are usually direction dependent, i.e. they respond differently to pushing and pulling. Many insects possess several attachment pads per leg. Typically, the distal ‘toe pads’ attach strongly when pulled towards the body, and readily detach when pushed away from it [4,6,9–12]. By contrast, proximal (tarsal) ‘heel pads’ are used for pushing away from the body [4,6]. In a recent study, we directly compared the attachment performance of distal ‘toe’ and proximal ‘heel pads’ of Indian stick insects (*Carausius morosus*, Phasmatodea, Sinety, 1901). While ‘toe pads’ generated large adhesive forces of up to one body weight, adhesion was negligible for ‘heel pads’ with forces smaller than *ca* 10% body weight [8]. However, ‘heel pads’ generated large friction that increased with load, and they were used solely under compression. Thus, ‘toe pads’ are true adhesive structures, whereas ‘heel pads’ appear to be ‘friction pads’ that generate high friction coefficients but little adhesion [8]. The division of labour between pads for pushing versus pulling as well as for friction versus adhesion allows insects to withstand both inward and outward shear forces, which occur during vertical climbing [13], while still retaining a mechanism for easy detachment. Moreover, it enables insects to restrict the use of delicate adhesive structures to situations where attachment is required [4,8,12].

**Figure 1.**
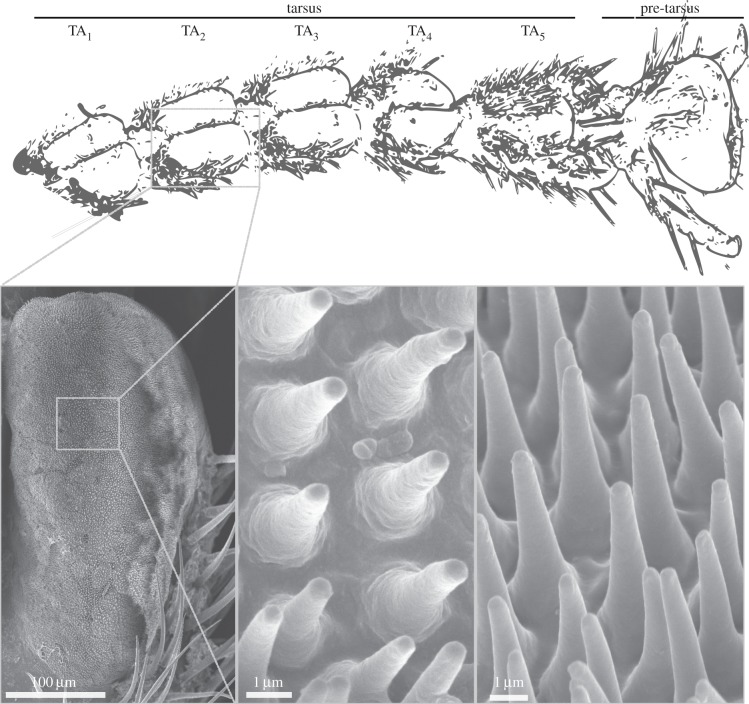
Morphology of the tarsus of *Carausius morosus* stick insects. The tarsal segments TA_1–4_ bear pairs of attachment pads (euplantulae) which are covered with conical cuticular outgrowths (acanthae).

The functional divergence between ‘heel pads’ and ‘toe pads’ is reflected in characteristic morphological differences. For example, distal and proximal pads in beetles usually consist of different seta types [6,14]. ‘Toe pads’ (arolia) of *Nauphoeta cinerea* cockroaches are smooth, whereas the surface of their ‘heel pads’ (euplantulae) is patterned by transverse ‘friction ridges’, which have a steeper slope on the distal side, enhancing pushing forces on rough surfaces [15]. The arolia of stick insects also have a smooth surface [8,16,17], but the euplantulae of many species are covered by short hair-like outgrowths (figure 1; [8,17,18]), which have been identified as acanthae (i.e. outgrowths of individual epidermal cells) in previous studies [19,20].

Superficially similar arrays of hairs (setae) are often found on pads that generate high adhesion, for example the distal pads of beetles, flies, spiders and geckos. Similar to many synthetic tacky materials, these fibrillar adhesive pads show strong adhesion, and as a result high friction coefficients; their friction forces can be virtually independent from load [12]. This stands in clear contrast to the properties of fibrillar ‘heel pads’ in stick insects [8]. Tarsal pads with arrays of conical acanthae occur in at least four insect orders: Phasmatodea, Mantophasmatodea, Plecoptera and Hymenoptera [20–23]. These structures may therefore represent convergent developments, suggesting that the ‘hairy’ morphology has functional advantages for their role as friction pads. What is the detailed function of these conical surface structures? Bußhardt *et al*. [18] measured friction and adhesion of different types of euplantulae (smooth versus covered by acanthae) present in two different stick insect species, and found that, only for the smooth euplantulae, friction was reduced on a rough compared with a smooth surface. Bußhardt *et al.* [18] suggested that the outgrowths are an adaptation to cope with surface roughness.

Here, we study the functional morphology of the fibrillar ‘heel pads’ of stick insects by focusing on the following questions: (i) How do ‘heel pads’ respond to load? (ii) Can the load-dependent increase in contact area fully explain the observed change in shear forces? (iii) What morphological characteristics are responsible for the combination of large friction coefficients and small adhesion?

## 2. Material and methods

### 2.1. Reflected-light microscopy

In order to investigate the influence of normal load on the real contact area of the ‘heel pads’ of Indian stick insects, the pad contact zone was studied at controlled loads using a Leica DRM upright microscope (Leica Microsystems Ltd, Heidelberg, Germany), with brightfield monochromatic epi-illumination from a mercury short-arc lamp (546 nm, HBO 103 W/2; Osram, Munich, Germany). Adult stick insects (*Carausius morosus*, Phasmatidae; body mass: 0.49 ± 0.09 g, mean ± s.d., *n* = 10) were taken from a laboratory colony fed with ivy and water *ad libitum*. Stick insects were slid into thin plastic tubes, and the dorsal side of one isolated leg was mounted on a piece of metal wire attached to the tube, so that the ventral side of the second or third pair of euplantulae was the highest point (for details, see [8]).

**Figure 2.**
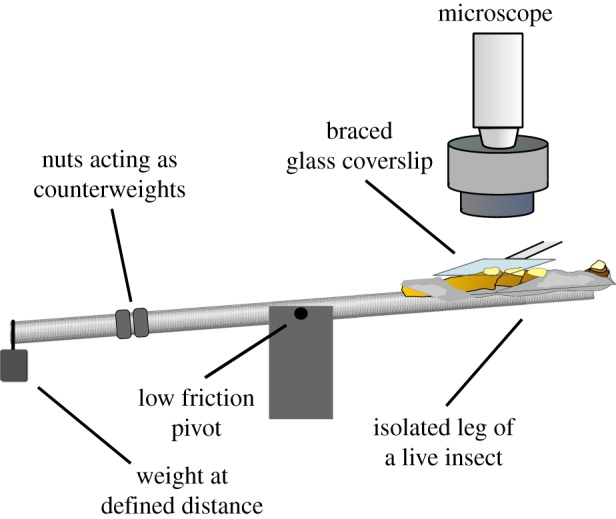
Schematic of the set-up used to study the influence of normal load on the surface contact of euplantulae. Stick insects were put into a thin plastic tube, and a single leg was attached to a piece of metal wire, so that the ventral side of the second or third pair of euplantulae was the highest point. The tube was attached to a threaded metal rod pivoted in the middle, and the additional weight of the insects was balanced using nuts that were screwed onto the rod as counterweights. Once balanced, weights were applied at the end of the metal rod to bring the pads in contact with a glass coverslip with a known normal load. (Online version in colour.)

As an electronic feedback control of normal force was not available in combination with our high-resolution microscope, the influence of normal force on the appearance of the contact zone was investigated with a ‘see-saw’ device, consisting of a threaded metal rod, pivoted at its centre (figure 2). A stick insect mounted in a plastic tube was attached to the metal rod. The additional weight of the insect was balanced by screwing nuts onto the rod as counterweights. Once balanced, weights corresponding to the normal force to be applied were added at a defined lever arm to bring the pad into contact with a glass coverslip mounted on a holder. Images at 0.2, 0.5, 1, 2 and 4 mN normal load (corresponding to around 5%, 10%, 25%, 40% and 80% body weight) were taken with a 5×, 20× or 100× oil immersion objective to measure (i) the projected contact area of the pad, (ii) the density of the acanthae in contact and (iii) the contact area of individual acanthae (for details, see below). These measurements were performed for 10 different insects. We also used a micro-manipulator to bring pads into contact with a glass coverslip, as this allowed us to apply small shear movements (≈50 μm) once the pad was in contact. Images and videos (10 fps) were recorded with a QICAM 10-bit monochrome camera (Qimaging, Burnaby, BC, Canada).

### 2.2. Data analysis and statistics

All contact area images were analysed using ImageJ v. 1.46a [24]. Projected contact area was measured by drawing the smallest polygon that still contained all acanthae in surface contact around one single euplantula. Acantha density (i.e. number of acanthae per unit contact area) was measured from images taken at random positions by binary conversion and subsequent use of the particle analysis routines implemented in ImageJ v. 1.46a. For the analysis of the density of acanthae, we analysed 18 075 ± 3288 μm^2^ per animal (mean ± s.d.), on average corresponding to around 25% of the projected contact area. The contact area of individual acanthae was then measured from images taken at the highest available magnification (100 × oil immersion × 1.6 video zoom), using binary conversion and particle analysis routines available in ImageJ v. 1.46a. The resolution of the light microscope is approximately ± 150 nm. The measurement error introduced by this physical limit scales with the circumference of the contact area. For the smallest measured circular contact area of around 0.12 μm^2^, this error is significant, around 75% of the contact area, but quickly reduces for larger contact areas. We verified that the results were reliable even for small contact areas by comparing several automated measurements with measurements carried out by hand. In total, we analysed the contact area of at least 250 acanthae per insect (on average 358 acanthae). In order to avoid pseudo-replication, statistical analysis was performed over the mean of the density/tip area per individual, resulting in an overall sample size of 10. We also measured the aspect ratio, i.e. the major axis of the contact zone divided by the minor axis for 243 acanthae selected to represent a wide range of different contact areas. These measurements were performed by drawing ellipses or polygons around individual contact areas by hand, as the automated aspect ratio measurements of ImageJ v. 1.46a appeared not to be reliable for the smallest contact areas observed in this study. Data for the friction of euplantulae at different normal loads are from reference [8]. Measurements were taken using a custom-made, two-dimensional strain-gauge force transducer. The shear resistance of a single pair of euplantulae, defined as the peak friction during a 2 mm slide on glass coverslips, was recorded at a sliding velocity of 0.1 mm s^−1^ (for more details, see [8,25]). During these measurements, the normal load was kept constant at either 1, 2 or 4 mN, using a motor-controlled 50 Hz force-feedback mechanism, and the projected contact area of the euplantulae was recorded simultaneously using reflected light. In order to investigate whether the changes in contact area observed in this study can explain the previously reported increase in friction, we calculated the real contact area *A*_R_ of the pad as the product of the apparent contact area *A*_A_, acantha density *N*_A_ and the mean contact area per acantha *A*_Ac_:2.1
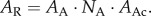


Friction data were corrected for *A*_R_ and statistically analysed with a repeated-measures ANOVA on ranks (Friedman's test), to account for the non-normality of the data. The influence of load on the different measures of contact area was analysed with a repeated-measures ANCOVA. In addition, we report the *η*^2^ effect size, which indicates the fraction of the variance accounted for by the normal force. Confidence intervals for *η*^2^ were calculated via the MBESS package in R [26–28]. Linear regression was conducted with a linear-mixed-model approach in order to account for the repeated-measures design. In order to provide a goodness-of-fit measure, we calculated both marginal and conditional *R*^2^, which indicate the variance explained by the fixed factor alone (marginal) and the fixed and random factors combined (conditional, for details on this method see [29]). Data reported in the text are given as mean ± s.d. unless otherwise indicated. Boxplots show the interquartile range and the median, whereas whiskers indicate 1.5× interquartile length. All statistical analyses were performed using R v. 3.0.1 [30]. All data are available from the Dryad digital repository (http://doi.org/10.5061/dryad.jj0kj).

## 3. Results

### 3.1. Contact area of euplantulae

**Figure 3.**
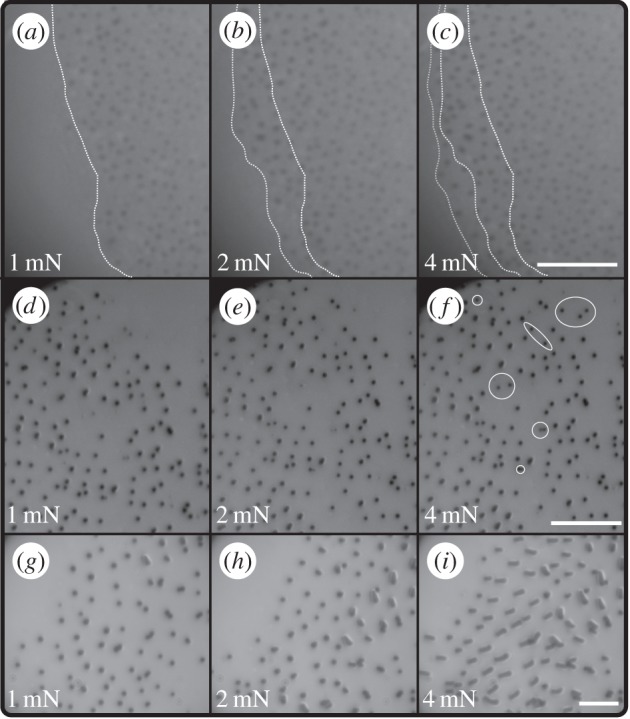
Reflected-light microscopy images illustrating the three hierarchical levels at which euplantulae contact area increased with load. Normal load increases from left to right from 1 to 2 to 4 mN. (*a–c*) First, larger loads increased the projected contact area. Dotted lines show the margin of the contact area for 1 (white), 2 (light grey) and 4 mN (dark grey). (*d–f*) Second, larger normal loads increased the density of acanthae in surface contact within the contact area itself. Some newly acquired contact points are highlighted by the ellipses in panel (*f*). (*g–i*) Third, higher normal loads induced a switch from tip contact to side contact of some acanthae. Scale bars, (*a–c*) 15 µm; (*d–f*) 10 µm and (*g–i*) 5 μm.

Reflected-light microscopy revealed that normal load increased the contact area of the euplantulae on three hierarchical levels (figure 3 and table 1). Higher normal loads increased (i) the projected contact area (figure 3*a–c*), (ii) the number of acanthae in contact within the projected contact area (figure 3*d–f*), and (iii) the contact area of individual acanthae, by inducing them to change from tip to side contact (figure 3*g–i*). All three mechanisms of contact area change were largely reversible, i.e. when the load was removed, the newly acquired contact area disappeared.

**Table 1.** Normal load influenced the contact area of euplantulae of Indian stick insects (*Carausius morosus*) at three levels. Given are the mean ± the margin of error (half the 95% confidence interval) as calculated with a *t*-distribution (*n* = 10).

load (mN)	projected contact area (mm^2^)	density (μm^−2^)	tip-contact area (μm^2^)
0.2	0.034 ± 0.007	0.071 ± 0.006	0.235 ± 0.031
0.5	0.061 ± 0.010	0.088 ± 0.010	0.292 ± 0.058
1	0.08 ± 0.013	0.107 ± 0.011	0.326 ± 0.059
2	0.092 ± 0.013	0.124 ± 0.011	0.404 ± 0.058
4	0.101 ± 0.014	0.132 ± 0.007	0.461 ± 0.073

### 3.2. Influence of load on projected contact area

**Figure 4.**
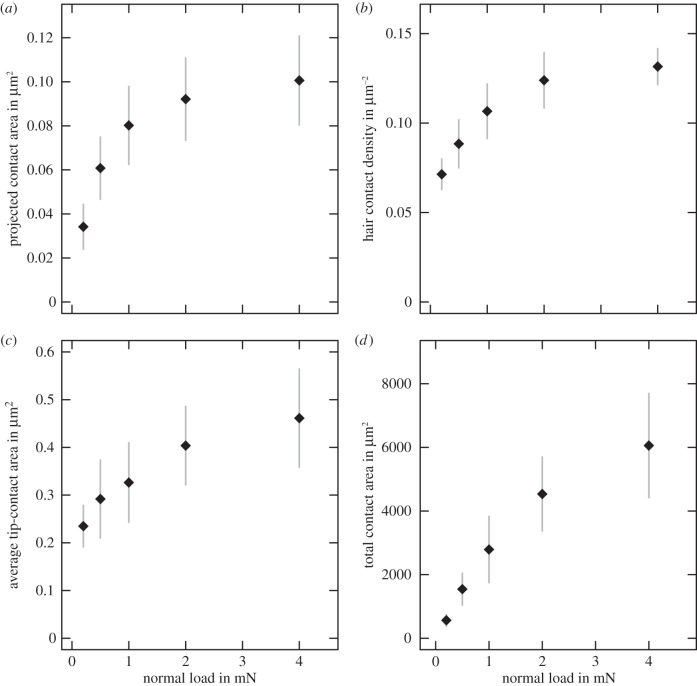
Normal load increased (*a*) the projected contact area (one pair of euplantulae), (*b*) acantha density and (*c*) the contact area of individual acanthae. (*d*) The overall increase in contact area, i.e. the product of (*a–c*). Shown are the mean ± s.d. (all *n* = 10).

Consistent with our previous findings [8], load significantly increased the overall euplantula area in surface contact (repeated-measures ANCOVA, *F*_1,39_ = 76.27, *p* < 0.001, *η*^2^ = 0.49 (95% CI: 0.28–0.63), *n* = 10). Projected contact area nearly tripled from the 0.2 load to the 4 mN load (figure 4*a* and table 1).

### 3.3. Influence of load on density

The density of acanthae in surface contact significantly increased with load (repeated-measures ANCOVA, *F*_1,39_ = 116.7, *p* < 0.001, *η*^2^ = 0.6 (95% CI: 0.41–0.71), *n* = 10; figure 4*b* and table 1). For all normal loads, the density appeared to be highest towards the distal–lateral edges. For small loads in particular (0.2 and 0.5 mN), the more proximal and central parts of the pad showed patches of contact surrounded by completely contact-free patches. The size of these non-contact patches continuously decreased with load. However, even for the highest normal load, some of these patches still existed, providing a possible explanation for why the effective mean density was below the acanthae density measured from scanning electron microscope (SEM) images (0.19 μm^−2^, [8]). However, the highest measured densities of individual areas were around 0.18 μm^−2^, indicating that, in some parts of the pad, all acanthae were in contact.

### 3.4. Influence of load on contact area of individual acanthae

**Figure 5.**
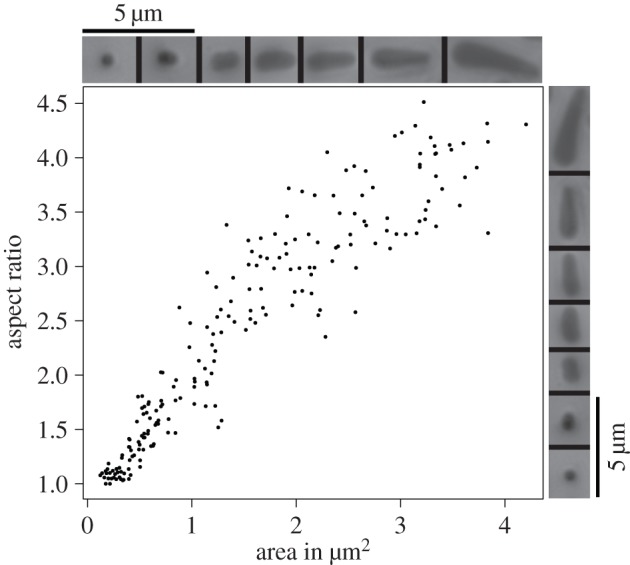
Relationship between aspect ratio (contact zone length per width) and contact area of individual acanthae. For larger contact areas, the aspect ratio of the contact area increasingly departed from 1, indicating bending of acanthae and thus side contact. The continuous aspect ratio range from 1 (circular tip contact) to 4.5 (full side contact), indicates that the transition from tip to side contact occurs gradually. The insets show representative contact area shapes for different aspect ratios per area.

Normal load significantly increased the contact area of individual hairs (repeated-measures ANCOVA, *F*_1,39_ = 124, *p* < 0.001, *η*^2^ = 0.47 (95% CI: 0.26–0.61), *n* = 10; figure 4*c* and table 1). At 0.2 mN, the contact zone of most acanthae was approximately circular (figure 5). With increasing load, individual acanthae bent significantly and made side contact, in particular in the lateral–distal zones of the contact area. The largest contact areas of acanthae in side contact (at 4 mN load) were around 1.30 ± 0.27 μm^2^ (*n* = 28 from three individuals), corresponding to a sixfold increase in contact area when compared with pure tip contact. Patches where acanthae were in side contact also appeared in the more proximal–central parts when the load was increased, but there always existed considerable areas with no acanthae in side contact. As a consequence, the mean contact area of the acanthae at 4 mN was only around 0.46 ± 0.10 μm^2^ (*n* = 10), despite some individual contact areas that were considerably larger.

When normal load was further increased to 8 mN (only measured for three animals), some acanthae appeared to make side contact over their full length, reaching contact areas of up to 4 μm^2^, nearly 20 times larger than tip-only contact (see insets in figure 5). Figure 5 shows the relationship between shape (aspect ratio) and magnitude of the contact area of acanthae over the range of different contact areas measured in this study. The contact zones changed continuously from circular tip contact (aspect ratio close to 1) to elongate side contact (aspect ratios up to 4.5), suggesting that acanthae gradually deform without apparent instabilities.

### 3.5. Overall increase in contact area

Figure 4*d* shows that real contact area increased by approximately an order of magnitude over the range of normal loads used in this study.

### 3.6. Influence of shear load

**Figure 6.**
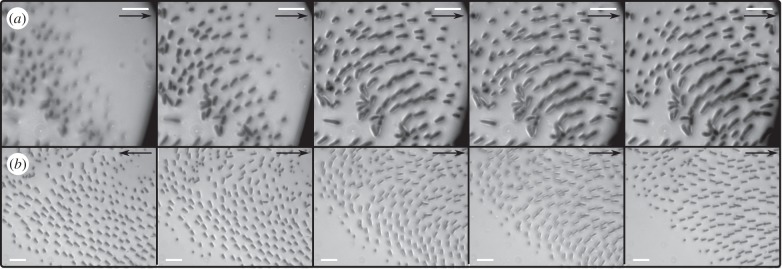
Effect of shear force on the contact of euplantula acanthae visualized by reflected-light microscope. Black arrows show the direction of the applied force. (*a*) Shear forces lead to a transition from tip to side contact. (*b*) When the direction of the shear force was reversed, individual acanthae changed orientation. The time interval between two consecutive images is 200 ms. Scale bars are 5 μm for all images.

The contact of individual acanthae was also sensitive to shear forces. Acanthae in side contact aligned with the direction of the applied force (figure 6*b* and the electronic supplementary material, videos S1 and S2). When a small shear movement was applied with the micro-manipulator, some of the acanthae, previously in tip-only contact, bent and made side contact (figure 6*a* and the electronic supplementary material, video S3).

### 3.7. Pad secretion

When euplantulae were detached, fluid droplets were left behind (see electronic supplementary material, figure S1). These droplets were comparable in size and spacing to the acantha tips, indicating that the acanthae themselves are covered by a secretion. The liquid was non-volatile, and remained stable over several minutes after the pad detached, similar to earlier observations on insect adhesive pads [31–34].

### 3.8. Influence of load on friction

**Figure 7.**
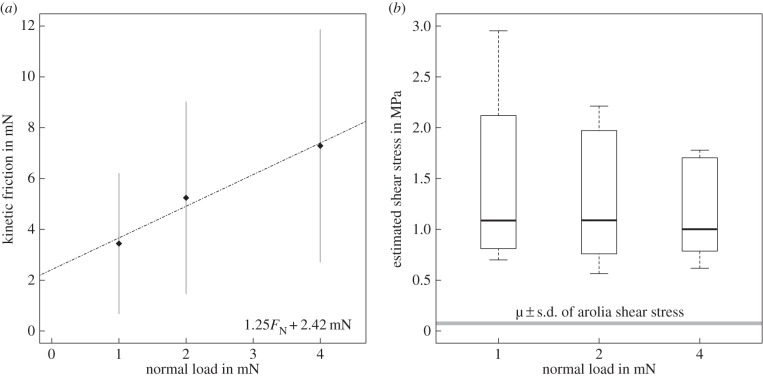
(*a*) Kinetic friction of a single pair of euplantulae of *Carausius morosus* over normal load. Shown are the medians and median absolute deviations (*n* = 10, taken from reference [8]). The dashed line is the result of an ordinary least-squares linear regression using only the medians for each normal force level. (*b*) The shear stress of the euplantulae was estimated by dividing the friction data by the mean real contact area as calculated with equation (2.1). The shear stress does not change significantly with load, indicating that the increase of friction in panel (*a*) is fully explained by the increase of contact area. The grey box in panel (*b*) shows the shear stress level of stick insect ‘toe pads’ (arolia) as measured in reference [8].

Figure 7*a* shows that friction increased significantly with normal load (Friedman's test, *χ*^2^ = 11.4, d.f. = 2, *p* < 0.01, *n* = 10), corresponding to effective coefficients of friction 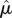 (friction per load) between 4 at 1 mN, and 2 at 4 mN load (data taken from reference [8]). However, friction corrected for the increase in real contact area, as calculated with equation (2.1), was no longer significantly influenced by normal load (Friedman's test, *χ*^2^ = 0.2, d.f. = 2, *p* = 0.90, *n* = 10; figure 7*b*). Thus, acanthae appear to generate a constant shear stress of 1.00 ± 0.49 MPa (median ± median absolute deviation, *n* = 10).

## 4. Discussion

Climbing insects must be able not only to attach safely, but also to detach rapidly and with little effort. Adhesive pads of insects show several adaptations that help them to combine these seemingly contradictory demands. For example, their contact area increases quickly and passively in response to unexpected detachment forces [35–37]. Adhesive forces can be controlled precisely and reversibly via shear forces [8], allowing easy detachment [4,12]. However, as smooth adhesive pads of insects have a very soft cuticle [16], it may be advantageous to limit their use to situations where adhesive forces are required, minimizing the risk of damage [38,39]. When legs are used during upright walking or below the centre of body mass during vertical climbing, their footpads only need to generate friction, as they are pressed against the surface, i.e. no force tends to detach the legs. Our results show that Indian stick insects possess ‘friction pads’ specialized for such situations. Analogous to adhesive pads, friction pads have properties allowing the insect to control the surface contact precisely and reversibly. Here, however, normal forces control friction forces (and not vice versa): euplantulae of stick insects respond to normal load with a fast increase in contact area.

### 4.1. Hierarchical load dependence of contact area

Euplantula contact area changed with load at three hierarchical levels. First, increasing normal load enlarged the projected contact area of the euplantulae (figure 3*a–c*). This is most likely explained by the curvature of the convex euplantulae themselves, as illustrated in figure 1. The projected contact area cannot exceed the pad's surface area, which for a pair of euplantulae is around 0.1 mm^2^ (measured from SEM images in reference [8]). Euplantulae under load asymptotically tended towards this maximum contact size as shown in figure 4*a*, and the full projected area was reached at a load of 4 mN (approx. 80% body weight).

**Figure 8.**
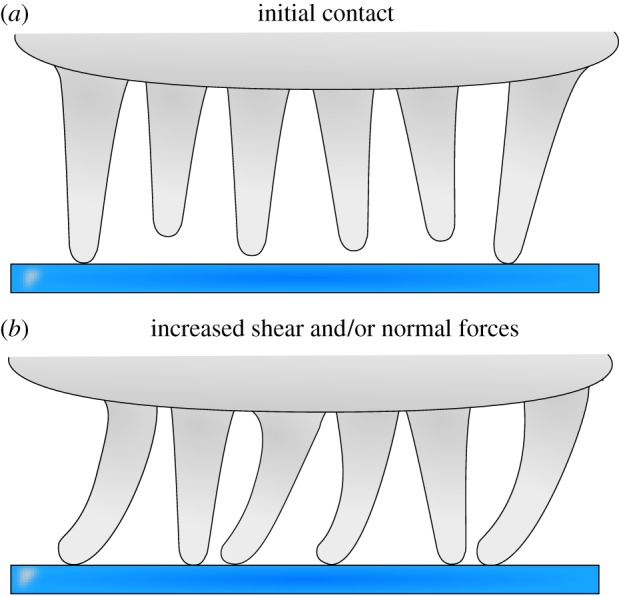
Schematic of the influence of normal and shear load on euplantulae. (*a*) For small loads, only a fraction of the acanthae are in surface contact. (*b*) If normal and/or shear loads are increased, acanthae in initial surface contact will bend, decreasing their effective length. Thus, more acanthae come into surface contact and contribute to shear resistance. (Online version in colour.)

Second, the density of acanthae in surface contact within the projected contact area increased with load (figure 3*d–f*), probably because the tips of the acanthae are not coplanar [8]. As a response to the increased load, individual acanthae may get compressed or bent, which reduces their effective length, thereby allowing other acanthae to come into contact (figure 8). Such an effect has been discussed previously for synthetic fibrillar arrays tested under shear conditions [40,41]. Bending of acanthae might be particularly easy at the periphery of the pad, where the orientation of acanthae follows the curvature of the pad, i.e. they are increasingly non-vertical, and thus the effective stiffness of the outgrowths may be reduced. This may also explain why both density and contact area appeared to be higher in this part of the pad contact area.

Third, light microscopy and video recordings showed that an increase in normal load induced a transition from tip to side contact for individual acanthae (figure 3*g–i*; for similar observations on technical adhesives, see [42–46]). Figure 5 suggests that this transition occurs after an initial phase of tip compression. For areas below approximately 0.3 μm^2^, the aspect ratio remains close to 1, indicating circular contact areas. Thus, there might be a critical load that induces the change from tip to side contact. Our findings show that acanthae in side contact can have a 5–20 times larger contact area than acanthae in tip contact, whereas higher normal forces increased the friction per projected contact area only by a factor of less than 1.5 [8]. A possible explanation for this discrepancy is that only a small fraction of acanthae were in side contact during the force measurements. Alternatively, it is likely that the shear forces measured at the smallest normal load were already sufficient to induce large amounts of side contact. Side contact is more easily achieved by a combined normal and shear load [43,45–48], because shear increases both the compliance of fibrillar structures and their tendency to buckle ([46,48–50], see also the electronic supplementary material, video S1). Hence, shear forces may increase not only the amount of side contact, but also the density of acanthae in surface contact, as a result of the effective reduction in length of the deflected fibres [40,41]. This is consistent with the observation that the optical contrast of the euplantulae contact area increased significantly with shear force, indicating a change in real contact area [8]. A further indication that we underestimated the real contact area of euplantulae during sliding is the estimated shear stress of around 1 MPa, which is nearly two orders of magnitude higher than that of the arolia and other previously recorded values for insect adhesive pads (figure 7*b* and [8,12]). Thus, our shear stress estimate should be seen as an upper limit.

All three mechanisms for the increase of contact area with load can be empirically described by power laws. The observed power-law dependence may arise from the varying curvature of the pad (for projected contact area), from the height distribution of acantha tips (for acantha contact density) and from the variation of side-contact length of individual acanthae with load (see the electronic supplementary material). As the load scaling coefficients for projected contact area, acantha density and acantha contact area are all numbers between 0 and 1 (table 2), the three hierarchical levels combine to a near-linear increase of the real contact area with load (figure 4*d* and table 2). This may be readily understood when equation (2.1) is rewritten as a function of normal force assuming power-law relationships and negligible adhesion,4.1
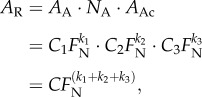
where *k_i_* are the slopes of the log–log plots of *A*_A_, *N*_A_ and *A*_Ac_ over load, respectively. The coefficients for projected contact area, acantha density and acantha contact area sum up to 0.802, relatively close to a linear relationship (table 2).

**Table 2.** Parameters of linear-mixed-model regressions on log–log data for all combinations of contact area measures and load. Given are the estimates of the intercept and slope along with their standard errors, as well as the conditional and marginal *R*^2^-values of the regression after [28].

parameter	intercept	scaling coefficient	conditional *R*^2^	marginal *R*^2^
projected contact area	−2.687 ± 0.073	0.362 ± 0.023	0.973	0.651
acanthae density	−16.097 ± 0.038	0.214 ± 0.009	0.986	0.735
acanthae contact area	−14.924 ± 0.062	0.226 ± 0.014	0.975	0.536
real contact area	−6.078 ± 0.093	0.802 ± 0.029	0.989	0.85

### 4.2. The origin of large coefficients of friction

In principle, generating friction is a trivial matter: moving any two bodies in contact relative to each other will result in some friction. In many cases, the magnitude of friction *F*_F_ depends linearly on the applied normal load *F*_N_, an empirical relationship commonly referred to as Amonton's law,4.2
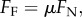
where *μ* is a proportionality constant, usually called the coefficient of friction. For most rigid materials, 0 < *μ* < 1. If the friction pads of insects were in this range, then at least one body weight of normal force would have to be applied to generate one body weight of friction, resulting in force vectors with a large angle to the substrate. This is in conflict with the tendency of sprawled-posture insects to align forces along the leg in order to minimize joint torques [51], i.e. force vectors usually have small angles to the substrate. Thus, it may be a significant advantage to possess friction pads with a friction coefficient greater than 1, as observed for the ‘heel pads’ of stick insects [8].

**Figure 9.**
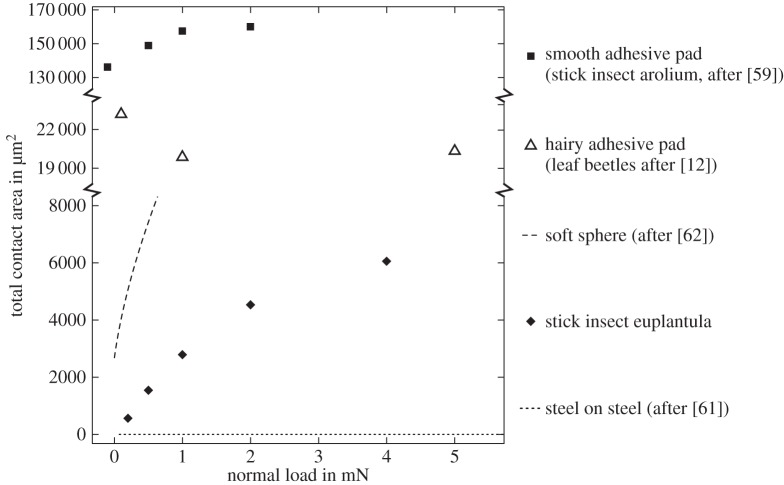
The change of real contact area with load for a number of different materials. Stick insect euplantulae show negligible contact area at near-zero loads, but respond to loading with a rapid increase of contact area. Adhesive pads, in turn, make complete contact at small or even negative loads. Effectively, euplantulae behave like rigid materials (dotted line) at small loads, but resemble soft materials (dashed line) at larger loads. For further details see text.

Friction coefficients *μ* > 1 are usually reported for soft materials where intermolecular adhesion can result in the significant contact area, even in the absence of external load (but see [52,53], for stiff materials that show large friction coefficients, but little adhesion). A simplified way to understand the effect of additional load owing to intermolecular adhesive forces on friction is to include adhesion into Amonton's friction law [54–56],4.3
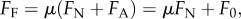


where *F*_F_, *F*_N_ and *F*_A_ are friction, normal and adhesion forces, and *F*_0_ = *μF*_A_ is the friction force at zero normal load, i.e. the intercept of a linear regression of friction over normal load. An ordinary least-squares linear regression of the median friction values of euplantulae as a function of normal load *F*_N_ yields *F*_F_ = 1.25*F*_N_ + 2.42 mN, suggesting the adhesion to be *F*_A_ = 1.94 mN. However, euplantulae show minuscule macroscopic adhesion in the absence of shear load [8,18]. Consistently, the results presented in figure 4 suggest that there is only very little contact area at zero load, in contrast to the behaviour of a soft material. Instead, we suggest that the large measured friction coefficients may be explained by a rapid initial increase in contact area with normal load, caused by the specific morphology of the euplantulae. In order to illustrate this point, we will in the following compare how real contact area increases with load for ‘friction pads’ versus both soft adhesives and rigid materials (figure 9).

There is ample evidence that friction is directly proportional to the real contact area between two bodies, i.e. materials appear to generate approximately constant shear stresses [57–60], consistent with our results for the ‘heel pads’ of stick insects. Equation (4.2) may thus be rewritten in terms of equation (4.1), multiplied by the shear stress *τ*4.4
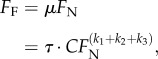
and thus4.5
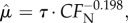
where 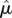 is an ‘apparent’ (i.e. load-dependent) friction coefficient. Equation (4.5) indicates that the friction coefficient for stick insect euplantulae decreases with load. Our friction measurements show that this is indeed the case, and the observed scaling coefficient of −0.46 (95% CI: −0.92−0.00) is consistent with equation (4.5). A similar decrease of the friction coefficient with load is seen in soft adhesive materials, which can make full contact at zero normal load. Biological adhesives such as the ‘toe pads’ of stick insects [8,12,25] also fall into this category, and not only smooth, but also fibrillar adhesive pads of insects may require soft and delicate tip structures for good adhesion [61]. These footpads can achieve full contact at zero normal load (figure 9). Such properties, however, would be undesired for ‘friction pads’, as they might increase the wear of pads, and hinder rapid detachment.

By contrast, contact at zero normal load and adhesion is effectively absent for rigid materials. They usually follow the classic friction law, i.e. friction is linearly proportional to load, and the friction coefficient is a constant independent of load. In this situation, equation (4.5) may be written as4.6
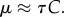


Here, the friction coefficient represents the product of the shear stress and the growth rate of real contact area with load, *C*. Measurements of contact area via conductance [62] suggest that *C* for finely ground steel on steel is extremely small, approximately 10^−6^ mm^2^ mN^−1^ (figure 9). For clean steel, *μ* ≈ 0.8, thus *τ* for steel on steel is *ca* 800 MPa. While the shear stresses we measured for euplantulae were two orders of magnitude smaller (around 1 MPa), their friction coefficient is significantly larger than that for steel. Thus, for euplantulae to exhibit 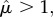 but no adhesion, *C* must be large, i.e. small changes in load must cause large changes in contact area. From a linear regression of *A*_R_ over load, we find empirically that *C* for euplantulae is *ca* 2.1 · 10^−3^ mm^2^ mN^−1^, three orders of magnitude larger than for steel on steel (figure 9). As an intermediate stage between the two extremes of very soft adhesives and rigid materials, figure 9 includes the prediction for a soft sphere of 1 MPa stiffness, a work of adhesion of 60 mN m^−1^ and a radius of 180 μm (calculated after [63]), i.e. comparable to the size of the euplantulae. Here too, contact area increases quickly with load, but there is significant adhesion. In terms of their friction performance, euplantula ‘friction pads’ combine properties from both soft adhesives and rigid materials: effectively, euplantulae behave like rigid materials at zero load, but like soft materials when load is increased.

### 4.3. The functional design of ‘frictional hairs’

Our estimate of the adhesive force of single acanthae (66 nN, see the electronic supplementary material) is comparable to the adhesion of individual spider setulae (41 nN) and gecko spatulae (10 nN) measured by atomic force microscopy [64,65]. An approximate comparison, assuming a contact area of around *π r*^2^ for the acanthae, yields an adhesion per unit area of ≈0.33 MPa for stick insect acanthae, 0.24 MPa for spiders and ≈0.25 MPa for gecko spatulae (assuming a rectangular shape of the gecko spatula of 200 × 200 nm; [64,65]). However, setules and spatulae are adhesive hairs [64,66], whereas pads covered with acanthae show negligible adhesion, but large coefficients of friction. What renders fibrillar structures adhesive, and what makes them ‘frictional hairs’?

We suggest that stick insect euplantulae show several morphological features that ensure minimal adhesion, which distinguish them from fibrillar structures used for adhesion.

First, the real contact area of the euplantulae is minimal at small loads, as the euplantulae are curved, the acanthae are not coplanar and the pointed tip geometry results in a low area coverage of acantha tips (less than 5%). This is in strong contrast to the fibrillar adhesive pads of some dipteran and coleopteran insects. For example, adhesive hairs in some flies have a comparable density [67], but a spatula tip width of ≈1–1.5 μm, resulting in an area coverage of approximately 10–20%. Adhesive hair arrays of *Gastrophysa viridula* beetles are highly planar; the whole enlarged tip of a seta can make full contact at zero normal load, and they show load-independent friction [12]. The real contact area of euplantulae, by contrast, is load dependent.

Second, in order to increase the real contact area, energy has to be invested to deform the pad and individual acanthae. We have observed such deformations directly using reflected-light microscopy. The elastic energy stored in the deformed pad and acanthae may partly be available to break the newly acquired contact area during detachment, decreasing the detachment force. Here, the tapered geometry of the acanthae may play an important role in allowing the tips of individual acanthae to rotate, generating the characteristic elliptical or lozenge-shaped contact patches evident in figure 3, while simultaneously preventing any sudden lateral buckling under the imposed compressive loads. The aspect ratio of the conical or tapered acanthae is small in comparison with that of adhesive hairs found in other arthropods (around 5 in comparison with 10–80; [68–70]), and, unlike some seta stalks, acanthae are not curved. Both morphological features dramatically reduce the lateral deformation of acanthae under compression, and thus their tendency to adhere laterally and their likelihood of undergoing sudden Euler buckling [71].

Third, even if some tips were still in contact at zero or negative load, their hemispherical geometry leads to stress concentration near the contact edges, thus reducing the peak adhesive force in comparison with other tip geometries commonly found in adhesive hairs, such as spatula- or mushroom-tipped fibrils [72,73]. In addition, the hemispherical tip impedes the shear sensitivity exhibited by pads that are used for adhesion by leading to concentric peeling [74].

Fourth, while the projected contact area of euplantulae and arolia (adhesive ‘toe pads’) is comparable, their aspect ratio (defined as width/proximal–distal length) differs strongly (≈1 versus more than 3 for euplantulae and arolia, respectively; see [8]). During detachment, stress will be concentrated at the peel-front and decay to zero over a characteristic distance *d* [75]. If the load is not equally distributed across the contact area, then attachment forces will be substantially smaller [76,77]. Attachment pads will usually detach along their proximal–distal axis, and, as a consequence of the small contact area aspect ratio of euplantulae, stresses during detachment are thus most likely to be concentrated in a small fraction of the contact area. A similar difference in aspect ratio between the most distal and the proximal pads has also been reported for *Tettigonia viridissima* bush crickets, which lack an arolium but possess a laterally widened distal euplantula (see figure 2 in [78]).

### 4.4. Comparison between ‘frictional hairs’ and ‘adhesive hairs’

It appears that the design criteria for acanthae and euplantulae are essentially the opposite of those for fibrillar adhesives; they are ‘designed’ to be poor adhesives (table 3). Several morphological features of pads, acanthae and their tips ensure that contact area can increase quickly with load, producing high friction coefficients, but detachment forces are negligible. Hence, euplantulae are friction pads that are of particular use in situations where insects need to generate friction, but no adhesion.

**Table 3.** Comparison between functionally relevant morphological features of ‘frictional hairs’ and ‘adhesive hairs’.

	frictional hairs	adhesive hairs	functional relevance
pads	convex	flat, hair tips coplanar	relationship between contact area and load
	as long as wide	wider than long	load sharing
	short	long	effective work of adhesion/compressive compliance
hairs	straight	curved	tensile compliance
	tapered	± cylindrical	storage of elastic energy in bending
	small	large	area coverage
hair tips	spherical/pointed	spatula- or mushroom-shaped	stress distribution

A similar performance has been reported for synthetic fibrillar structures consisting of stiff polypropylene microfibres or of carbon nanotubes with curly entangled tops [52,53]. Insect pads with arrays of acanthae may provide valuable biological models for further improving high-friction, low-adhesion surfaces. At the same time, incorporating features of natural friction pads such as the conical geometry into the design of such synthetic structures may allow us to investigate their functional significance in more detail.
